# Search for the fourth elusive haplotype of the *APO E2/E3/E4* polymorphism and its potential effect on AD risk

**DOI:** 10.1002/alz.089595

**Published:** 2025-01-03

**Authors:** Asma Naseer Cheema, Elizabeth C Lawrence, Narges Zafari, Kang‐Hsien Fan, Muhammad Muaaz Aslam, Vibha Acharya, Margaret A Bedison, Eleanor Feingold, M. Ilyas Kamboh

**Affiliations:** ^1^ School of Public Health, University of Pittsburgh, Pittsburgh, PA USA

## Abstract

**Background:**

The common *APOE2/E3/E4* polymorphism, the strongest risk factor for Alzheimer’s disease (AD), is determined by two‐site haplotypes at codons 112 (Cys>Arg) and 158 (Arg>Cys), resulting into six genotypes. Due to strong linkage disequilibrium between the two sites, 3 of the 4 expected haplotypes (E2, E3, E4) have been observed and extensively studied in relation to AD risk. Compared to the most common haplotype of E3 (Cys112 – Arg158), E4 (Arg112 – Arg 158) and E2 (Cys112 – Cys158) haplotypes are determined by a single‐point mutation at codons 112 and 158, respectively. The fourth haplotype having mutations at both sites (Arg112 – Cys158) has been reported only as an incidental finding in five kindreds. To our knowledge, no systematic search has been done to determine its distribution in many AD and non‐AD subjects. The study aimed to search for the elusive fourth haplotype, elucidate its frequency and contextual association with AD.

**Method:**

A DNA fragment of 177bp from subjects with the *APOE* 2/4 genotype was subcloned with single deoxyadenosine (A) overhang on 3’ end that was amplified and ligated into 4kb PCR^TM^ II‐TOPO vector with single 3’ deoxythymidine(T) residue then transformed into chemically competent cells (*E.coli* DH5aTM ‐T1^R^) to construct the phased haplotype clones. Plasmid DNA was extracted from successful clones and sequenced. We also confirmed the haplotype phasing by whole‐ genome sequencing.

**Result:**

We extracted *APOE* genotype data on more than 14,000 AD and non‐AD subjects previously genotyped in our laboratory and identified 344 subjects with the *APOE* 2/4 genotype who are compound heterozygotes by carrying mutations at both sites. Thus far, subcloning followed by single‐stranded sequencing in 313 of the 344 samples have yielded phased haplotypes and the results indicate that the E4 and E2 mutations are present on opposite chromosomes. Thus, no example was observed where both the E4 and E2 mutations are on the same chromosome (Arg112 – Cys158). We are in the process of determining the haplotype phases in the remaining samples.

**Conclusion:**

No example of the elusive fourth haplotype was found in our large AD case‐control dataset, suggesting it might have a minimum effect, if any, on AD risk.

